# Correction: Evaluation of the Efficacy of the HaeNaem Zero Bone Loss Kit in Indirect Sinus Lift Using Osseodensification

**DOI:** 10.7759/cureus.c200

**Published:** 2024-11-21

**Authors:** Rachel Changrani, Amod P Patankar, Swapna A Patankar, Pranjali Kulkarni, Amisha Sharma

**Affiliations:** 1 Oral and Maxillofacial Surgery, Bharati Vidyapeeth (Deemed to be University) Dental College and Hospital, Pune, IND; 2 Oral Pathology and Microbiology, Bharati Vidyapeeth (Deemed to be University) Dental College and Hospital, Pune, IND

This article has been corrected to add a citation and corresponding reference to the following statement in the conclusions: "Moreover, when compared to Densah burs, the OD and indirect sinus lift performed with HaeNaem drills offer a more favorable risk-to-benefit ratio [12]."

12. Sharma R, Patankar AP, Patankar S, Pawar SR, Tiwari V, Khan N: Effect of indirect sinus lift using osseodensification technique on osseous dimensions at the antral floor in dental implants placement. J Oral Health Dent. 2023, 6:454-459.

